# Ameliorative effect of cotreatment with the methanolic leaf extract of *Urtica dioica* on acute kidney injury induced by gentamicin in rats 

**Published:** 2020

**Authors:** Saeed Hajihashemi, Mahboubeh Ahmadi, Ali Chehrei, Fatemeh Ghanbari

**Affiliations:** 1 *Department of Physiology, Faculty of Medicine, Arak University of Medical Sciences, Arak, Iran*; 2 *Department of Pathology, Faculty of Medicine, Arak University of Medical Sciences, Arak, Iran*; 3 *Department of* *Pharmacology, Islamic Azad University, Arak Branch, Arak, Iran *

**Keywords:** Gentamicin, Cotreatment, Nephrotoxicity, Urtica dioica, Nephroprotective, Rat

## Abstract

**Objective:**

Effects of cotreatment with *Urtica dioica* (UD) methanolic leaf extract on gentamicin (GM)-induced acute kidney injury were evaluated in rats.

**Materials and Methods:**

Male Wistar rats (n=32) were separated into four groups. Gentamicin (100 mg/kg/day, IP) was injected for eight days with or without UD methanolic extract (200 mg/kg/day, gavage).  The renal blood flow (RBF) and systolic blood pressure of rats were recorded. Concentration of creatinine, blood urea nitrogen (BUN), sodium, and potassium and osmolarity were measured in the urine and plasma samples. Oxidative stress level was determined by assessment of the levels of antioxidant power (FRAP) and lipid peroxidation (MDA) in the renal tissue. The renal injury and histopathological changes in the kidney were determined by microscopic evaluations.

**Results:**

Administration of UD extract along with GM, compared to GM group, significantly decreased the amounts of plasma creatinine and BUN, urinary sodium excretion, fractional excretion of sodium and potassium, and MDA levels but significantly increased creatinine clearance, urine osmolarity, renal blood flow and FRAP levels.

**Conclusion:**

The cotreatment of UD extract can attenuate renal injury of GM by reduction of oxidative stress, lipid peroxidation, and oxygen free radicals. The potential nephroprotective effects of UD extract are probably mediated via its antioxidant and anti-inflammatory activity.

## Introduction

The aminoglycoside antibiotic gentamicin (GM) is prescribed extensively against infections, particularly aerobic Gram-negative bacteria. Renal toxicity and ototoxicity are principal toxic effects of GM. Gentamicin induces renal toxicity in 10–20 percent of the cases in the course of therapy which can lead to acute kidney injury (Nagai and Takano, 2004 ▶; Randjelovic et al., 2017 ▶; Lopez-Novoa et al., 2011 ▶). Nephrotoxicity of GM demonstrates a nonoliguric acute renal failure with declining renal blood flow (RBF) and disorders of urinary concentration and dilution. Therefore, it causes hypoosmolar urinary output and increases in plasma creatinine after several days of treatment (Beauchamp and Labrecque, 2001 ▶; Nagai and Takano, 2004 ▶; Randjelovic et al., 2017 ▶). Nephrotoxicity of GM is induced following its accumulation in the epithelial cells through the endocytic pathway in the renal proximal tubules (S1 and S2 segments). It induces tubular dysfunction accompanied by necrosis and apoptosis of the epithelium cells (Martínez-Salgado et al., 2007 ▶; Nagai and Takano 2014 ▶; Randjelovic et al., 2017 ▶). 

Previous studies showed that GM nephrotoxicity is associated with excess production of reactive oxygen species (ROS) in the kidney. Gentamicin suppressed antioxidant defense enzymes such as superoxide dismutase (SOD), glutathione peroxidase (GSH-Px) and catalase (CAT) that scavenge ROS in the kidney (Baliga et al., 1999 ▶; Martínez-Salgado et al., 2007 ▶). Oxidative stress, tubular necrosis, inflammation and reduction of glomerular filtration rate are induced by ROS such as superoxide anion, hydrogen peroxide, and hydroxyl radicals in the kidney. The glomerular filtration rate (GFR) decreases through the production of vasoconstriction substances by ROS, therefore antioxidants can prevent the decline of renal blood flow (Nath and Norby, 2000 ▶; Randjelovic et al., 2017 ▶).

ROS cause renal cell damage, necrosis of tubular epithelial cells through lipid peroxidation, protein denaturation and DNA damage (Edson and Terrell, 1999 ▶; Servais et al., 2005 ▶). ROS activate NFκB that initiates the inflammatory process (Bledsoe et al., 2006 ▶; Tugcu et al., 2006 ▶; Tavafi and Ahmadvand, 2011 ▶).

The imbalance between the formation of ROS and antioxidant protection causes renal cellular injury (Quiros et al., 2010 ▶). Previous studies used antioxidants for treatment or attenuation of GM-induced renal toxicity (Safa et al., 2010 ▶; Hajihashemi et al., 2017 ▶). 

*Urtica dioica *(UD) is a medicinal herb which belongs to the Urticaceae family, with highly effective medicinal compounds concentrated in leaves (Joshi et al., 2014 ▶). *U. dioica *is an herbal medicine consumed traditionally for urinary tract infections, bladder problems, kidney stones, prostate enlargement, coughing, hair growth, Alzheimer's disease, allergic reactions, inflammatory diseases such as asthma, bronchitis, bursitis, gingivitis, gout, rheumatoid arthritis, and osteoarthritis and diabetes (Halder and Sharma, 2017 ▶). This plant has antioxidant, antimicrobial, anti-inflammatory and hypolipidemic properties (Haghju and Almasi, 2015 ▶). *U. dioica *extract contains glycosides, tannins, phenolic compounds, flavonoids, alkaloids and proteins (Safarinejad, 2006 ▶; Joshi et al., 2014 ▶; Hajihashemi et al., 2017 ▶). 

Antioxidant properties of this plant are due to the existence of phenolic compounds. In addition, the anti-inflammatory property depends on ingredients like adenine, nicotinamide, and synephrine. Vasodilatory properties of UD are related to the vasorelaxation effect of nitric oxide and blocking of calcium channel activity (Upaganlawar et al., 2006 ▶; Halder and Sharma, 2017 ▶). 

Previous studies in our laboratory demonstrated that post-treatment with UD methanolic extract for two days after kidney injury has therapeutic effects on GM renal toxicity in rats (Ahmadi et al., 2018 ▶).

An important goal of the present study was to examine the effects of cotreatment with UD methanolic leaf extract for eight days on GM induced nephrotoxicity in rats. 

## Materials and Methods

**Treatment of animals**

Experiments were done in 32 male albino Wistar rats (200-250 g) which had free access to water, and normal pellets *ad libitum*. Animals were acclimatized at stable laboratory temperature at 23±2ºC with 12 hr light-dark periods.

This scientific study was approved by Research Ethics Committee of Arak University of Medical Sciences. Ethical approval number:1394.87. 

All animal care and procedures were performed based on the moral codes and guidelines. 

**Experimental Design**

Experimental rats were randomly separated into four experimental groups of 8 rats each: 

Group 1: Received normal saline intraperitoneally (0.5 ml/day) for 8 days.

Group 2: Received GM intraperitoneally (100 mg/kg/day; Alborz Darou Co, Iran) for 8 days.

Group 3: Received normal saline intraperitoneally (0.5 ml/day) and UD methanolic extract (200 mg/kg/day; oral gavage) for eight days. 

Group 4: Received GM intraperitoneally (100 mg/kg/day) and UD methanolic extract (200 mg/kg/day; oral gavage) for eight days. 

After the treatment period, animals’ urine was collected for 24 hr by metabolic cages. The tail-cuff method was used for measuring the systolic blood pressure (AD Instruments, Australia) (Hajihashemi et al., 2017 ▶).

**Measurement of renal blood flow**

The left renal artery and vein were dissected. Measurement of renal blood flow was done from the left kidney artery using transonic flowmeter and PowerLab data acquisition system for 30 min (Ahmadi et al., 2018 ▶).

**Biochemical analysis**

Rat abdominal aorta blood sampling was collected using heparinized syringe. To separate plasma from samples, the blood was centrifuged at 10,000 rpm for 10 min (Eppendorf, Germany). The plasma was kept frozen at –20ºC and then used for biochemical analyses.  

The right kidney was used for biochemical experiments. The homogenization of the right kidney was performed in the solution of potassium phosphate buffer (0.1 M, pH 7.4) (Heidolph homogenizer Silent crusher-M, Donau, Germany). The centrifugation of the kidney homogenate was done at 10,000 rpm for 5 min. The supernatant of homogenate was used for the determination of the lipid peroxidation levels (Hajihashemi et al., 2018 ▶). 

The concentration of sodium, potassium, creatinine, and urea and osmolarity were determined in the urine and plasma samples. The concentration of creatinine [Cr] and urea [BUN] was measured by an autoanalyzer (Selectra XL, Netherlands). Sodium and potassium concentration was determined by a flame photometer (SEAC-FP20, Italy). Furthermore, total osmolality was measured using the Gonotec osmometer (Osmomat-030, Germany) (Ameen et al., 2011 ▶). 

The following equation was used for calculation of creatinine clearance rate, absolute and relative excretions of potassium and sodium:

1) Creatinine clearance rate (μl/min/gkw) = (V°/1000×U_Cr_)/P_Cr _(Calculation of GFR. V°: urine volume in 1-minute, U_Cr_: urinary creatinine concentration_)_.

2) Absolute excretion of sodium U_Na_V˚ (μmol/min/gkw)=(V°×U_Na_)/1000(Calculation of total sodium excretion. V°: urine volume in 1-minute, U_Na:_ urinary sodium concentration).

3) Absolute potassium excretion U_K_V˚(μmol/min/gkw)=(V˚×U_K_)/1000(Calculation of total potassium excretion. V°: urine volume in 1-minute, U_K_: urinary potassium concentration). 

4) Fractional excretion of sodium FE_Na_= (U_Na_×P_Cr_)/(P_Na_×U_Cr_) ×100 (Calculation of the percentage of the sodium filtered by the kidney. U_Na_***: ***urinary sodium concentration, U_Cr_: urinary creatinine concentration, P_Cr_: plasma creatinine concentration, P_Na_: plasma sodium concentration).

5) Fractional excretion of potassium FE_K_= (Uk×P_Cr_)/(Pk×U_Cr_) ×100 (Calculation of the percentage of the potassium filtered by the kidney. U_K_: urinary potassium concentration, U_Cr_: urinary creatinine concentration, P_Cr:_ plasma creatinine concentration, P_K_: plasma potassium concentration).

**UD extract preparation**

UD leaves were collected from the mountains of Kurdistan province in Iran from May to June. The expert of ethnobotany authenticated UD Plant. The leaves of UD were rinsed with water, and then dried up in the shadow at room temperature. The powder of leaves was prepared by a laboratory blender. The extraction of UD was performed by methanol. The powdered plant material (500 g) was soaked in methanol for 72 hr with intermittent shaking. The resulting suspension was passed through Whatman filter paper No. 2. The filtrate was passed through a new Whatman filter paper. The filtrate solution was evaporated at 60°C using a rotary evaporator. The extract was incubated for 24 hr at 60°C so that its methanol would be entirely evaporated and dry plant extract was obtained (Kataki et al., 2010 ▶).

**Malondialdehyde (MDA) levels measurement**

The amount of MDA was evaluated as the indicator of lipid peroxidation in the kidney. The homogenization of the kidney tissue was done in the phosphate buffered solution (at 1/10 W/V). The homogenate (100 µl) was added to a reaction mixture (200 µl of sodium dodecyl sulfate (SDS) 8.1%, 1500 µl of thiobarbituric acid 0.8%, 1500 µl of acetic acid 20% (pH 3.5) and 700 µl distilled water. The mixture was heated for 1 hr at 95°C and then centrifuged at 3000 rpm for 10 min. After separation of the supernatant, the absorbance was measured at 532 nm by a spectrophotometer (SpectroLab 7500 UV, England) (Ohkawa et al., 1979 ▶).

**Ferric reducing antioxidant power (FRAP) measurement**

Measurement of the total antioxidant activity was performed using Benzie and Strain method. The reduction of ferric (Fe^+3^) to ferrous (Fe^+2^) in the presence of Tripyridyl-S-Triazine (TPTZ) indicates the antioxidant power. The concentration of blue TPTZ-Fe^+2^ complex measured by spectrophotometer. The FRAP reagent was prepared by mixing ferric chloride, TPTZ, acetate buffer and distilled water. Homogenized tissue samples (50 μl) were mixed with the fresh FRAP reagent (1.5 ml) in tubes and after 4 min, the absorbance was measured at 593 nm by a spectrophotometer (SpectroLab 7500 UV, England). The amount of FRAP were shown as mmol/ gram kidney weight (gkw) (Benzie and Strain, 1996 ▶).

**Histological evaluations**

The fixation (10% buffered formaldehyde solution) and paraffin- embedding of kidney tissue were done respectively. Renal tissue in the paraffin blocks was cut at 5- micron thickness. Histological examination was done for tissue sections mounted on slides that were stained with hematoxylin and eosin. The evaluation of morphometric analysis and imaging was done by a specialist of pathology. The pathological changes such as increase of Bowman’s space, the percentage of tubular and glomerular cell injuries, high red blood cells count (RBCs) in glomeruli, downfall of tubular cells, existence of cast proteins in the lumen, necrosis of tubular cells and vacuolation were evaluated.

On the basis of pathological changes observed in the glomerular and renal parenchyma, the severity of injuries was scored as follows: "grade 0" (no injury), "grade 1" (less than 25% injury), "grade 2" (injury of 25-50%), "grade 3" (injury of 50-75%), and "grade 4" (injury of 75-100%) (Al-Shabanah et al., 2009 ▶).

**Statistical analysis**

The data are reported as mean±standard error of the mean (S.E.M.). Data analyses were done by SPSS software (version 20, Chicago, USA). The data was statistically analyzed by One-way analysis of variance (ANOVA), Kruskal-Wallis, and Tukey’s test. Multiple comparisons were done by the Dunnett’s tests. The probability values less than or equal to 0.05 were considered statistically significant.

## Results

**UD extract effects on the systolic blood pressure and renal blood flow (RBF)**

**Figure 1:**
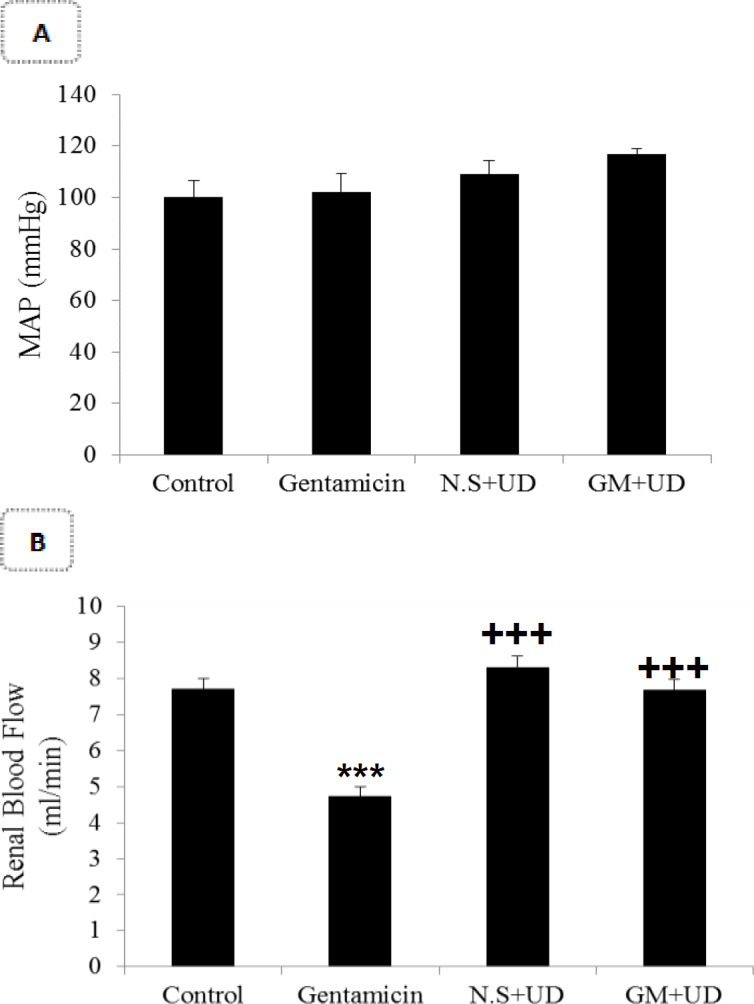
Comparison of A) Renal blood flow and B) Systolic blood pressure among the groups

The results showed that cotreatment of UD extract and GM in comparison to the control group did not significantly change the systolic blood pressure (Figure 1.A). Gentamicin in comparison to the control group significantly reduced RBF (4.35±0.21 ml/min vs. 7.70±0.28 ml/min; p<0.001). Concurrent treatment with UD extract and GM increased RBF as compared to the GM group (7.68±0.23 ml/min vs. 4.35±0.21 ml/min; p<0.001, Figure 1.B). There were no significant differences in the systolic blood pressure among the groups.

**Effects of UD extract on absolute sodium (U**_Na_ **V** ^o^ **) and potassium (U**_k_ **V** ^o^ **) excretions, relative sodium (FE**_Na_ **) and potassium (FE**_k_ **) excretions and clearance of creatinine (C**_Cr_ **)**

In the present study, administration of GM, compared to the control group, significantly enhanced plasma creatinine concentration (0.57±.02 mg/dl vs. 2.39±0.15 mg/dl; p<0.001), and BUN (20.50±0.96 mg/dl vs. 92.00±2.44 mg/dl p<0.001) but significantly decreased urine creatinine (54.3±1.7 mg/dl vs. 27.50±1.30 mg/dl; p<0.001) and BUN (140.6±16.70 mg/dl vs. 39.20±5.80 mg/dl; p<0.001) indicating renal damage. The results indicated that GM significantly decreased creatinine clearance (0.4±0.02 ml/min/kg vs. 1.30±0.03 ml/min/kg; p<0.001) in comparison with the control. There were significant differences in the creatinine clearance between the UD group and the control group (1.70±0.10 ml/min/kg vs. 1.30±0.03 ml/min/kg; p<0.001). The concurrent treatment of rats with GM and UD extract significantly increased creatinine clearance (0.4±0.01 ml/min/kg vs. 1.30±0.10 ml/min/kg; p<0.001). 

In this study, results showed that GM significantly increased relative excretion of sodium compared to the control group (5.1±0.4 % vs. 0.4±0.03 %; p<0.001). The relative excretion of sodium in the UD group (0.4±0.05%) was not significantly different from the control group. The co-treatment of rats with GM and UD extract indicated a significant reduction in the relative excretion of sodium in comparison to the GM group (5.1±0.4% vs. 0.7±0.1%; p<0.001).

Compared to the control group: ***p<0.001 show significant differences. Compared to the GM group: +++p<0.001 show significant differences. Results are expressed as the standard error of the mean (SEM) for eight rats in each group.

In the GM group, the relative excretion of potassium significantly increased in comparison with the control group (139±7.7% vs. 34.6±2.5%; p<0.001). In the UD extract-treated rats in comparison to the control group, the relative excretion of potassium (36.2±3.70%) was not significantly changed. The rats treated with GM and UD extract, compared to the GM group, significantly reduced the relative excretion of potassium (53.50%±7.50% vs. 139±7.7% p<0.001). The absolute excretion of potassium and sodium was not significantly different among the groups (Table 1).

**Table 1:** Comparison of creatinine clearance (C_Cr_), absolute (U_Na_V^o^) and relative (FE_Na_) excretions of sodium and absolute (U_k_V^o^) and relative (FE_k_) excretions of potassium

**C**_cr_ **(ml/min/kg)**	**U**_Na_ **V** ^o^ **(mmol/min/kg)**	**U**_k_ **V** ^o^ **(mmol/min/kg)**	**FE**_Na_ **%**	**FE**_K_ **%**	**Parameters **
					**Groups**
1.29±0.03	0.77±0.05	1.99±0.16	0.43±0.03	34.63±2.49	**Control**
*** 0.39±0.01	*** 2.78±0.22	2.65±0.11	*** 5.06±0.42	*** 139.00±7.71	**Gentamicin**
+++ 1.44±0.06	+++ 0.97±0.14	2.16±0.27	+++ 0.43±0.05	+++ 36.24±3.66	**NS+UD**
+++ 1.35±0.09	+++ 1.43±0.18	+++ 2.98±0.38	+++ 0.73±0.10	+++ 53.52±7.53	**Gentamicin+** **UD** **concurrent) ** **)**

Compared to control group: ***p<0.001 show significant differences.

Compared to the gentamicin group: +++p<0.001 show significant differences.

Results are expressed as the standard error of the mean (SEM)for eight rats in each group.

**Effects of UD extracts on urinary concentration of creatinine ([Cr]**_u_ **), potassium ([K]**_u_ **), sodium ([Na]**_u_ **), and urea ([BUN]**_u_ **), and osmolality (Osmolu**_u_ **)**

In the GM group, urinary sodium concentration significantly increased in comparison to the control group (81.3±1.8 μmol/ml vs. 55.7±2.9 μmol/ml; p<0.001). The urinary concentration of sodium (65.2±3.9 μmol/ml) was not significantly different between the UD and control group. The urinary concentration of sodium was significantly lower in the GM and UD extract group in comparison to the GM group (67.30±1 μmol/ml vs. 81.3±1.8 μmol/ml; p<0.001). 

In the GM group, the urinary potassium concentration was significantly lower than that of the control group (77.80±3 μmol/ml vs. 140.5±2.1 μmol/ml; p<0.001). In rats treated with GM and UD extract, urinary potassium concentration was significantly higher than that of the GM group (139.2 ±1.6 μmol/ml vs. 77.80±3 μmol/ml; p<0.001). In the GM-treated rats, urinary creatinine concentration was significantly lower than the control group (12.85±5 mg/dl vs. 41.42±4.5 mg/dl; p<0.05). In comparison to the control group, in the UD extract treated rats, urinary creatinine concentration did not significantly change (58.5±1.5 mg/dl vs. 54.3±1.7 mg/dl). Urinary creatinine concentration in rats treated with GM and UD extract, was significantly lower than the GM group (27.5±1.3 mg/dl vs. 58±2.2 mg/dl; p<0.001). 

Urinary BUN concentration was significantly lower in the GM-treated rats compared to the control group (39.2±5.80 mg/dl vs. 140.60±16.70 mg/dl; p<0.001). In addition, there was no significant difference in urinary urea concentration between the UD extract treated rats (120.20±11.90 mg/dl) and control groups (140.60±16.70 mg/dl). In rats treated with GM and UD extract, urinary BUN concentration was significantly higher in comparison to the GM group (127.20±11.10 mg/dl vs. 39.20±5.80 mg/dl; p<0.001).

In the present experiment, concurrent administration of UD extract and GM in comparison to the GM group urine osmolality (1218.70±56.40 mOsm/KgH2O vs. 553±48.80 mOsm/KgH2O; p<0.001), significantly increased (Table 2).

**Table 2.** Comparison of urinary concentrations of sodium ([Na]_u_), potassium ([K]_u_), creatinine ([Cr]_u_), and urea ([BUN]u), and osmolality (Osmol_u_) among the groups

**Osmol**_u_ **(mOsm/kgH2O) **	**[BUN]**_u_ **(mg/dl) **	**[Cr]**_u_ **(mg/dl) **	**[K]**_u_ **(μmol/ml) **	**[Na]**_u_ **(μmol/ml) **	**Parameters**
					**Groups**
1290.0±81.3	140.6±16.7	54.3±1.7	140.5±2.1	55.7±2.9	**Control**
*** 553.0±48.8	*** 39.2±5.8	*** 27.5±1.3	*** 77.8±3.0	*** 81.3±1.8	**Gentamicin**
1212.9±47.4	120.2±11.9	58.5±1.5	143.1±1.2	65.2±3.9	**UD+NS**
+++ 1218.7±56.4	+++ 127.2±11.1	+++ 58.0±2.2	+++ 139.2±1.6	+++ 67.3±1.0	**Gentamicin** **UD** **(concurrent) **

Compared to the control group: ***p<0.001show significant differences.

Compared to the gentamicin group: +++p<0.001 show significant differences

Results are expressed as the standard error of the mean (SEM)for eight rats in each group.

**Effects of UD extract on plasma concentrations of creatinine ([Cr]**_p_ **), potassium ([K]**_p_ **), sodium ([Na]**_p_ **), and urea ([BUN]**_p_ **), and osmolality (Osmol**_p_ **)**

In the present experiment, GM significantly increased the plasma concentration of creatinine (2.3±0.10 mg/dl vs. 0.6±0.02 mg/dl p<0.001) and BUN (92±2.4 mg/dl vs. 20.5±1 mg/dl; p<0.001) in comparison to the control group. UD extract treatment alone did not change the plasma creatinine concentration (0.6±0.03 mg/dl vs. 0.6±0.02 mg/dl) and BUN (21.20±1.20 mg/dl vs. 20.5±1 mg/dl) in comparison to control values. However, elevations in the plasma BUN (27.90±1.30 mg/dl vs. 92±2.4 mg/dl; p<0.001) and creatinine concentration (0.9±0.10 mg/dl vs. 2.3±0.10 mg/dl; p<0.001) were significantly reduced in rats concurrently treated with GM and UD extract in comparison to the GM group indicating reductions in GM-induced nephrotoxicity.

Significant differences were not seen in the plasma sodium concentration among GM group and different groups. 

Compared to the control group, plasma potassium concentrations did not change in the GM group (4.8±0.1 μmol/ml vs. 4.40±0.1 μmol/ml) and UD extract group (4.50±0.1 μmol/ml). Plasma concentration of potassium was not significantly different between UD extract+GM group and the GM group. (4.6±0.10 μmol/ml vs. 4.8±0.1 μmol/ml). Osmolality of plasma did not show significant differences among different groups (Table 3).

**Effects of UD extract on the** **malondialdehyde (MDA) and FRAP levels in the renal tissue**

**Figure 2:**
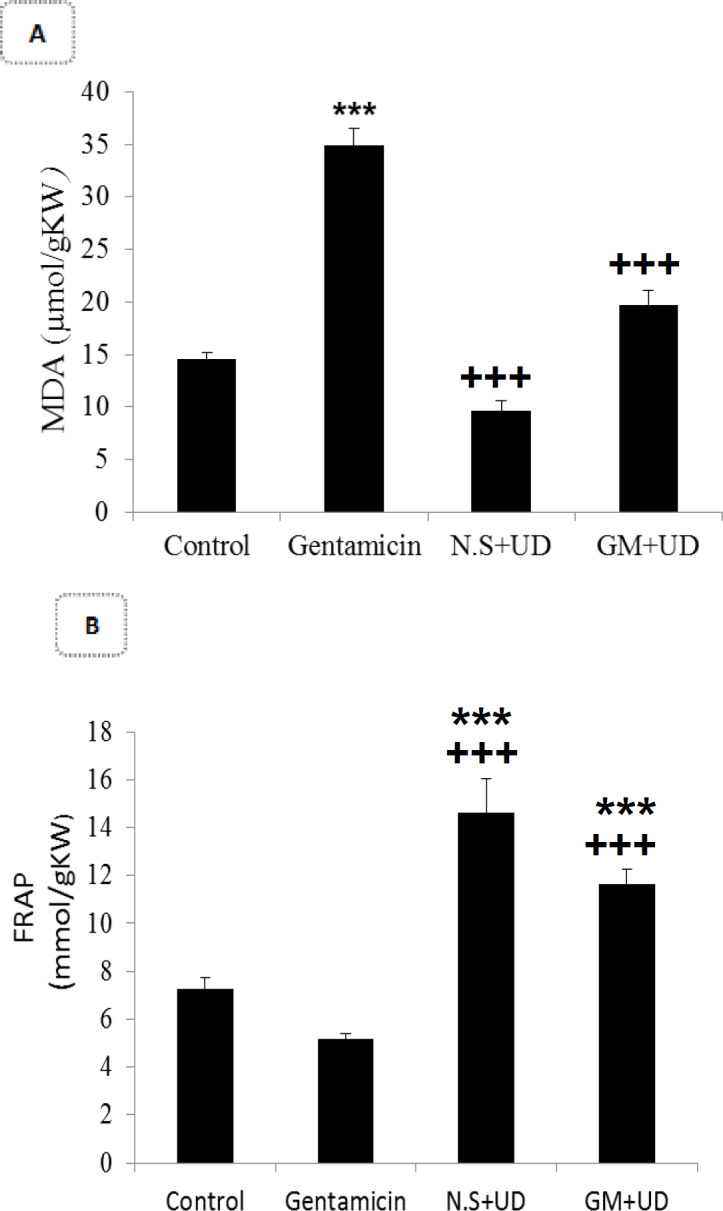
Comparison of A) MDA and B) FRAP levels among the groups.

In the GM group, MDA level significantly increased in comparison to the control group (31.77±2.04 μmol/gkw vs. 14.53±0.67 μmol/gkw; p<0.001). In the UD extract group, MDA level was not significantly different from that of the control group. However, in the GM+UD extract-treated rats, MDA level significantly declined in comparison to the GM group (11.4±1.03 μmol/gkw vs. 31.77±2.04 μmol/gkw; p<0.001; Figure 2A). In the GM group, FRAP level in the renal tissue was significantly lower compared to the control group (5.32±0.41 mmol/gkw vs. 7.27±0.44 mmol/gkw; p<0.001). In the rats concurrently treated with gentamicin and UD extract, the FRAP level was significantly higher than the GM group (14.78±0.99 mmol/kgw vs. 5.32±0.41 mmol/gkw; p<0.001; Figure 2B). 

**Table 3:** Comparison of plasma concentrations of sodium ([Na]_p_), potassium ([K]_p_), creatinine ([Cr]_p_), and urea ([BUN]_p_) and osmolality (Osmol_p_).

**Osmol**_p_ **(mOsm/kg H**_2_ **O) **	**[Cr]**_p_ **(mg/dl)**	**[BUN]**_p_ **(** **mg/dl** **)**	**[K]**_p_ **(μmol/ml) **		**Parameters**
					**Groups**
287.0±3.1	0.6±0.02	20.5±1.0	4.45±0.1	140.7±1.3	**Control**
292.6±3.7	*** 2.3±0.1	*** 92.0±2.4	4.8±0.1	138.5±0.5	**Gentamicin**
286.6±2.4	+++ 0.6±0.03	+++ 21.2±1.2	4.5±0.1	145.0±1.2	**NS+UD**
291.7±3.3	+++ 0.9±0.1	+++ 27.9±1.3	4.6±0.1	140.0±0.9	**Gentamicin+** **UD** **concurrent) ** **)**

Compared to the control group: ***p<0.001 show significant differences.

Compared to the gentamicin group: +++p<0.001 show significant differences.

Results were expressed as the standard error of the mean (SEM) for eight rats in each group.

Compared to the control group: ***p<0.001 show significant differences. Compared to the gentamicin group: +++p<0.001 show significant differences. Results are expressed as the standard error of the mean (SEM) for eight rats in each group. Results are expressed per gram kidney weight (gkw).

**Effects of UD extract on histological changes**

**Figure 3:**
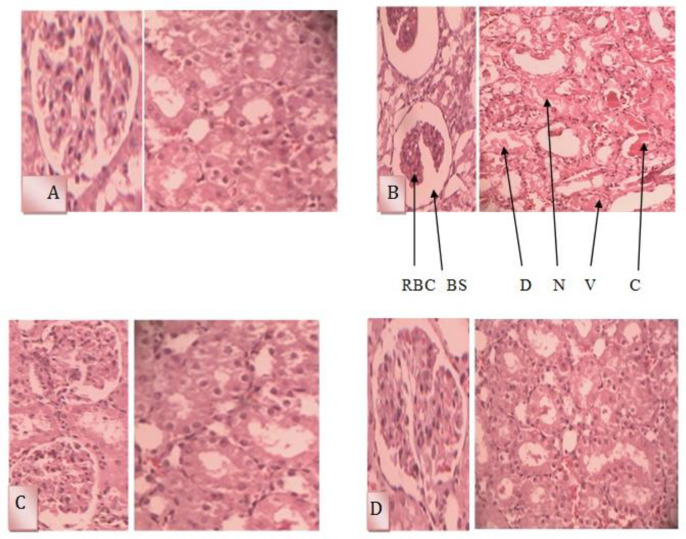
Comparison of renal histological between different groups

Extensive renal damage was observed in the GM-treated rats. Microscopic analysis of the kidney tissues indicated tubular cells' necrosis (grades 4; p<0.001), production of intraluminal protein casts (grades 3; p<0.001), renal tubular cells' vacuolization (grades 4; p<0.001) increase in space of Bowman’s capsule (grades 4; p<0.001), decreasing RBCs count (grades 3; p<0.001) and dispersion of cells into the lumen of the tubule (grades 3; p< 0.001; Figure 3, Table 4).

Following concurrent treatment of rats with GM and UD extract, the renal tissue injury was significantly decreased in comparison to the GM group. The UD extract caused significant decreases in cellular necrosis (grade 1; p<0.001), decrement of the Bowman’s space (grade 1; p<0.001), generation of protein casts within the lumen of the tubule (grade 1; p<0.001), dispersion of cells into the lumen of the tubule (grade 1; p<0.001) and vacuolization of renal epithelial cells (grade 2; p<0.001) and enhancement of the number of RBCs in the glomeruli (grade 1; p<0.001). In addition, the UD extract group (grade 0) and control group (grade 0) had a normal appearance (Table 4 and Figure 3).

**Table 4:** Comparison of necrosis level, protein casts, cell scattering, vacuolation, reduced number of red blood cells, increased Bowman’s space, and total glomerular injury

**Total glomerular injury**	**Increased Bowman’s space**	**Reduced number of red blood cells**	**vacuolization**	**Cell scattering**	**Formation of protein casts**		**Parameters**
							**Groups**
0	0	0	0	0	0	0	**Control**
*** 4	*** 4	*** 3	*** 4	*** 3	*** 3	*** 4	**Gentamicin**
+++ 0	+++ 0	+++ 0	+++ 0	+++ 0	+++ 0	+++ 0	**UD+NS**
*** +++ 1	*** +++ 1	*** +++ 1	*** +++ 2	*** +++ 1	*** +++ 1	*** +++ 1	**Gentamicin+** **UD** **(concurrent) **

Compared to the control group: ***p<0.001 show significant differences.

Compared to the gentamicin group +++p<0.001 show significant differences.

Results were expressed as the standard error of the mean (SEM) for eight rats in each group.

A- Control group with glomerular and normal tubular structure(×40); B- Gentamicin group with tubular cell necrosis, formation of protein casts inside the tubule lumen, cells scattering into the tubule lumen, vacuolation of tubular cells, increased Bowman’s space, and reduced number of red blood cells in glomerulus(×40); C- UD extract group with normal glomerular and tubular structures (×40); D- Concurrent treatment with gentamicin and UD extract showed reduced tubular cell necrosis, formation of protein casts inside the tubule lumen, cell scattering (×40), vacuolization of tubular cells, increased Bowman’s space, and increased number of red blood cells in glomerulus (×40). 

RBC: Red Blood Cells, BS: Bowman’s space, N: Necrosis, C: Intratubular cast, D: Downfall, V: Vacuolization.

## Discussion

This study indicated that co-treatment with UD extract and GM significantly increased creatinine clearance and RBF compared to the GM group. The results of this study showed the ameliorative effect of UD extract in GM-induced nephrotoxicity. 

Similar to the previous research (Martínez-Salgado et al., 2007 ▶; Nagai and Takano, 2014 ▶; Randjelovic et al., 2017 ▶), in this study, GM caused acute kidney injury as indicated by an increase in plasma urea and creatinine concentrations and increased urinary sodium and potassium excretion. Additionally, acute kidney injury induced by GM was verified by a decline in clearance of creatinine and excretion of urea in urine with histological injury evidence (Lopez-Novoa et al., 2011 ▶). In this study, GM treatment produced glomerular and tubular effects and significantly increased plasma creatinine and BUN concentration. The impairment of renal function indicated by increases in BUN and plasma creatinine.

Clearance of creatinine was used to evaluate the GFR. Gentamicin via glomerular effects reduced GFR. It caused mesangial cell contraction, neutralization of negative charges of glomerular filtration barrier, and apoptosis of mesangial cell (Martínez-Salgado et al., 2007 ▶). 

Our findings indicated that MDA level significantly increased in the GM-treated rats, which signified renal damage and necrosis. Elevated MDA level indicated more lipid peroxidation and involvement of free radicals in GM nephrotoxicity. The most important renal action of GM is the production of ROS such as hydroxyl radical and superoxide anion (Walker and Shah 1988 ▶). Increased ROS production is associated with damages in proximal tubule cells and necrosis of these cells. Subsequently, free radicals affect glomerular mesangial cells, cause contraction of these cells, reduce ﬁltration coefﬁcient (Kf) and consequently decline glomerular filtration rate (Polat et al., 2006 ▶).

In a previous study, it was shown that the antioxidant compounds such as resveratrol could increase GFR by inhibiting mesangial cells' contraction. Creatinine clearance was increased due to the elimination of free radicals (Morales et al., 2006 ▶). UD extract, because of having phenolic compounds such as caffeic and malic acid, has antioxidant and free radicals scavenging properties (Halder and Sharma, 2017 ▶). In this study, administration of UD extract inhibited oxidative stress induced by GM as shown by a decline in plasma BUN and Cr concentration in treated rats.

In this study, GM significantly decreased urine osmolarity compared to the control group. In the previous study, it was shown that GM significantly decreased the expression of aquaporin water channels (AQP-2) in rat renal medullary epithelial cells. Therefore, GM- induced disorders in urinary concentrating and dilution might be related to decreased AQP-2 expression in the inner and outer of renal medulla (Lee J et al., 2001 ▶). In this study, concurrent treatment with GM and UD extract increased urine concentration to the level of the control group. Because of the antioxidant components present in the UD extract, ROS production was reduced that prevented cells injuries and subsequently maintained the capacity of kidney in the urinary concentrating (Morales et al., 2006 ▶; Halder and Sharma, 2017 ▶).

 In this study, blood pressure did not vary significantly among the groups. In this study, similar to results of previous studies GM increased renal vascular resistance and reduced RBF (Klotman and Yarger., 1983 ▶; Hajihashemi et al., 2017 ▶). Reduction in RBF can be due to activating tubuloglomerular feedback (TGF) a protective mechanism preventing from losing electrolytes after tubular injuries (Lopez-Novoa et al., 2011 ▶). This feedback adapts after about 24 hr via the generation of vasoconstrictors like endothelin-1, and thromboxane A2 that can cause reduction of RBF (Valdivielso et al., 1999 ▶; Papanikolaou et al., 1992 ▶; Randjelovic et al., 2017 ▶).

Gentamicin-induced vasoconstriction and tubular injury was mediated by the generation of ROS, thus antioxidant components can play a protective role in GM-induced renal injuries. In previous studies, it was shown that trans-resveratrol has an antioxidant property and a nephroprotective effect on GM nephrotoxicity (Morales et al., 2002 ▶; Morales et al., 2006 ▶). 

Based on the results of this research, concurrent treatment with GM and UD extract significantly increased the renal blood flow compared to the GM group. It is possible that phenolic compounds have key roles in this effect (Halder and Sharma, 2017 ▶). In previous studies, it was shown that UD extract caused heart protection against ischemia/ reperfusion (I/R) injury due to its phenolic compounds (Shackebaei et al., 2010 ▶). Phenolic compounds having a hydroxyl group, can scavenge free radicals. In another *in vivo* study, it was shown that UD extract had vasorelaxant effects through increased nitric oxide production (Testai et al., 2002 ▶). Also, in rats treated with UD, RBF increased compared to the control group but it was not significant. 

In GM-treated rats, the urinary sodium and potassium excretion was increased that caused higher FENa and FEK. Consistent with the previous studies, FENa and FEK significantly increased subsequent to treatment with GM (Gowrisri et al., 2012 ▶; Hajihashemi et al., 2017 ▶). Previous studies showed that GM caused inhibition of Na^+^/K^+ ^ATPase activity in the basolateral membrane. Similarly, GM inhibited Na^+^/H^+^ exchanger and Na^+^/p_i_ cotransporter in the apical membrane (Williams et al., 1984 ▶; Sorribas et al., 2001 ▶). An increase in sodium and water inside the cells leads to cell swelling, cellular necrosis and increased sodium and potassium ion excretion (Banday et al., 2008 ▶; Park et al., 2010 ▶). 

In this study, the co-treatment with GM and UD extract significantly reduced ion excretion. Therefore, the antioxidant property of UD extract acts to reduce free radical and prevents tissue damage. The UD extract has an ameliorative effect against GM- induced plasma electrolyte disturbance.

In this study, concurrent treatment with GM and UD extract reduced lipid peroxidation and increased FRAP levels in the renal tissue. UD extract administration could decrease cell damages induced by GM because of its known antioxidant activity that inhibited the production of ROS. In another study, it was shown that administration of UD extract prevents tourniquet-induced oxidative stress in muscle (Cetinus et al., 2005 ▶). It was also shown that UD extract improved the antioxidant capacity and reduced reactive oxygen radicals in rats with hepatic injury induced by ischemia-reperfusion (Kandis et al., 2010 ▶). 

Treatment with UD extract reduced MDA levels in liver tissue of rats treated with tetracycline due to its antioxidant properties (Özen and Korkmaz, 2003 ▶; Kanter et al., 2005 ▶). Active compounds of UD extract were able to regulate endogenous enzyme such as glutathione reductase (GR), glutathione peroxidase (GPx), superoxide dismutase (SOD) and catalase (CAT) (Özen and Korkmaz, 2003 ▶).

In the present study, histological analysis showed that GM caused necrosis of epithelial tubular cells, formation intraluminal protein casts, vacuolar generation, and vascular congestion, increasing space of Bowman’s capsule and decreasing number of RBCs in the glomerulus. Histopathological findings of renal tissue indicated that concurrent treatment with UD extract and GM could be considered a potential protective approach to prevent tubular, vascular and glomerular damage. 

The UD extract has anti-inflammatory properties due to antioxidant and anti-inflammatory compounds like caffeic acid, malic acid, nicotinamide, and adenine (Halder and Sharma, 2017 ▶). In other studies, it was shown that the nuclear factor -kappa B (NF*k*B) is involved in nephrotoxicity induced by GM (Tugcu et al., 2006 ▶; Ozbek et al., 2009 ▶). Gentamicin caused an increase in NF*k*B via ROS production. The NF*k*B activates the expression of many inflammatory cytokines (Tugcu et al., 2006 ▶). Thus, the UD extract is able to inhibit inflammatory mediators such as NF*k*B and tumor necrosis factor-alpha (TNF_α_) (Riehemann et al., 1999 ▶; Yilmaz et al., 2014 ▶). 

According to our findings, co- treatment with UD extract had ameliorative effects on GM nephrotoxicity in rats. The method of treatment and duration of administration of eight days, possibly produce different results compared to the findings of the previous study. The nephroprotective effect of UD extract is , at least in part, related to its antioxidant compounds and anti-inflammatory characteristics; however, exact pathways involved in this effect need further experimental and clinical studies.
